# Association between prenatal androgens and cord blood androgens, a path analysis

**DOI:** 10.1038/s41598-022-25531-5

**Published:** 2023-01-07

**Authors:** Hui Xu, Qian Wang, Ting Li, Yuanyuan Wang, Ruiyao Cao, Xingwang Peng, Rongying Yao, Hui Han, Rui Zhou, Lianguo Fu

**Affiliations:** 1 Department of Child and Adolescents Health, School of Public Health, Bengbu Medical College, 2600 East Sea Avenue, Bengbu, 233030 Anhui China; 2 The Third People’s Hospital of Bengbu City, Bengbu, Anhui China; 3 Maternal and Child Health Care Center of Maanshan, Maanshan, 243000 Anhui China; 4 Department of Pediatric, The First Affiliated Hospital of Bengbu Medical College, Bengbu, 233004 Anhui China

**Keywords:** Developmental biology, Physiology

## Abstract

To determine association paths between prenatal androgens and cord blood androgens. The concentrations of T, FT, DHT, DHEA and SHBG in prenatal venous blood and cord blood were measured in 342 pregnant women and their neonates. The association paths between these hormones in prenatal and cord blood were revealed using Pearson correlation, multiple linear regression and path analysis. CB-T, CB-FT and CB-DHT in male neonates were higher than those in female neonates. In male and female neonates, P-FT was lower than CB-FT; however, P-DHT and P-SHBG were higher than CB-DHT and CB-SHBG, respectively. P-DHEA was lower than CB-DHEA in female newborns. In male neonates, there were association paths of P-T → CB-T → CB-FT → CB-DHT, P-T → CB-FT → CB-DHT, P-T → P-FT → CB-FT → CB-DHT, P-T → P-DHT, CB-DHEA → CB-DHT, CB-DHEA → P-DHT, and CB-DHEA → P-DHEA. In female neonates, there were association paths of P-T → CB-T → CB-FT → CB-DHT, P-T → P-FT → CB-FT → CB-DHT, P-T → P-FT → P-DHT, P-T → P-DHT, P-DHEA → P-DHT, CB-DHEA → P-DHEA, and CB-DHEA → CB-FT. There were differences in the T, FT and DHT concentrations in cord blood between male and female neonates and in the FT, DHT, DHEA, and SHBG concentrations between prenatal and cord blood. P-T and P-FT concentrations were positively associated with CB-T and CB-FT concentrations, while CB-DHEA concentration was positively associated with P-DHEA concentration.

## Introduction

It has become increasingly evident that androgens are not only necessary precursors for estrogen synthesis but also play direct roles in pregnancy^1^. Previous studies have shown that maternal androgens play roles in the regulation of the hypothalamic‒pituitary‒adrenal (HPA) axis in the fetus^2^. Exposure to hyperandrogens during pregnancy can induce anxiety-like behaviors in offspring^3^. Higher maternal free testosterone in early pregnancy was related to abnormal reproduction of male newborns^4^. Studies in rodent models showed that elevated androgen levels during pregnancy affected fetal growth^5^.

Androgens in pregnant women mainly include testosterone (T), free testosterone (FT), dihydrotestosterone (DHT), and dehydroepiandrosterone (DHEA). In the circulation, approximately 98% of T exists in its bound form, and only approximately 2% is free. According to the free hormone hypothesis, FT is the only form of T that is able to bind to its receptor or be converted into DHT to diffuse into cells and exert biological effects^6^. DHEA is a precursor of estrogen and androgen and combines with sulfates to form dehydroepiandrosterone sulfate (DHEAS). The main physiological function of sex hormone binding globulin (SHBG) is to specifically bind and transport hormones^7^.

The placenta participates in the biosynthesis and metabolism of steroids and regulates the exchange between the maternal and fetal compartments. However, the placental barrier prevents direct contact of maternal and fetal blood^8^. Placental aromatase is traditionally thought to protect the fetus from increased maternal androgen levels, but animal research contradicts this hypothesis^9^. Svensson^10^ noted that T is a lipophilic agent and can pass through the placenta. This study showed that maternal–fetal hormone transfer may be unidirectional, with hormones only passing from the mother to the fetus^11^. Cohen-Bendahan et al.^12^ reported that FT can cross the placental barrier from the fetal side into the maternal circulation. An in vivo study using deuterium-labeled DHEA(S) given to the fetal side in the perinatal period demonstrated that DHEA(S) originating from the fetal adrenal gland was transferred to the mother through the placenta^13^. The affinity of SHBG for T and DHT may change during pregnancy^14,15^. Due to the influence of various hormone, genetic and metabolic factors during pregnancy, there is a complex relationship between prenatal and cord blood androgens. Sex hormones of pregnant women and their newborns may interact with each other through the placental barrier^16^, while the metabolic environment of the intrauterine fetus may permanently change fetal development and may even affect the physiological state and behavior of adulthood through epigenetic development planning, resulting in pathological changes and a series of childhood and adult diseases. Therefore, knowledge of maternal–fetal hormonal relationships is needed. Irreversible damage to the fetus may certainly occur if these interactions are not taken into account. The purpose of this study was to determine the relationship paths between prenatal androgens and cord blood androgens to provide a theoretical basis for reducing the adverse birth outcome of newborns and promoting the early prevention of childhood diseases.

## Materials and methods

### Subjects

A total of 342 neonates and their mothers were effectively recruited in a grade 3 A hospital. The inclusion criteria for pregnant women were natural conception, singleton gestation, clear gestational age, no chromosomal disorders, no major illnesses, no intellectual problems, no psychiatric disorders, complete information on routine examinations during pregnancy, and signed informed consent form. Meanwhile, informed consent forms for the neonates' were signed by their mothers. This study was approved by the Medical Ethics Committee of the Bengbu Medical College ([2018] No. 015). All experiments were performed in accordance with relevant guidelines and regulations.

### Blood sample collection

Professional nurses collected 4 ml of venous blood from pregnant women before delivery (within 2 h of being sent to the delivery room or operating room); 3 ml of cord blood was collected during delivery (after delivery of the fetus and before delivery of the placenta). Using a high-speed centrifuge, the blood samples were centrifuged (3000 r/min, 15 min) to separate the serum on the day of blood collection, and the sera were stored in the refrigerator at − 80 °C for later unified detection of sex hormones.

### Hormone detection

The concentrations of T (ng/dl), FT (pg/ml), DHT (pg/ml), DHEA (ng/ml) and SHBG (nmol/l) in prenatal venous blood and cord blood were detected. The concentrations of serum T and SHBG were measured using chemiluminescence immunoassay (Roche 602 Chemiluminescence Autoanalyser). The concentrations of serum FT, DHT and DHEA were measured using radioimmunoassay methods (GC-2016 radioimmunometer).

### Statistical analysis

**Figure 1:**
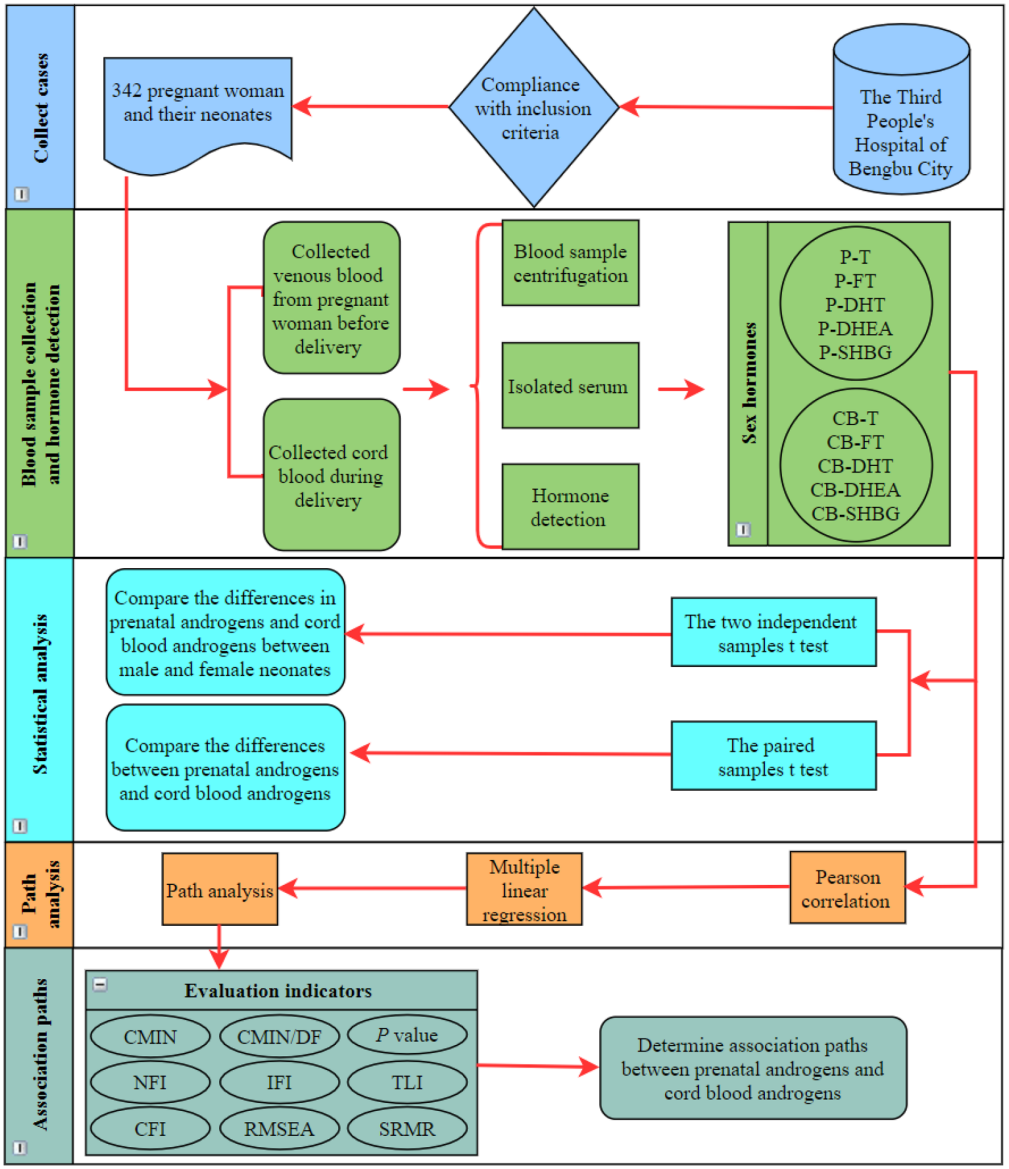
Flow chart of this study. Notes: *P-T* prenatal testosterone, *P-FT* prenatal free testosterone, *P-DHT* prenatal dihydrotestosterone, *P-DHEA* prenatal dehydroepiandrosterone, *P-SHBG* prenatal sex hormone binding globulin, *CB-T* cord blood testosterone, *CB-FT* cord blood free testosterone, *CB-DHT* cord blood dihydrotestosterone, *CB-DHEA* cord blood dehydroepiandrosterone, *CB-SHBG* cord blood sex hormone binding globulin, *CMIN* Chi-square, *CMIN/DF* Chi-square/degree of freedom, *NFI* normed fit index, *IFI* incremental fit index, *TLI* Tucker–Lewis index, *CFI* comparative fit index, *RMSEA* Root Mean Square Error of Approximation, *SRMR* Standardized Root Mean Residual.

SPSS 23.0 software was used for the data analysis. The quantitative data are described as the mean (SD). The two independent samples *t* test was used to compare the differences in prenatal androgens and cord blood androgens between male and female neonates. The paired samples *t* test was used to compare the differences between prenatal androgens and cord blood androgens. Pearson correlation was used to analyze the correlations between prenatal androgens and cord blood androgens. Furthermore, multiple linear regression was used to analyze the associations between prenatal androgens and cord blood androgens. Finally, path analysis was used to fit the relationship path between prenatal androgens and cord blood androgens. The maximum likelihood method was used to estimate model parameters. The 95% CI of the path coefficient was calculated by the bootstrapping method. The fitting of the model was assessed using the following parameters: Chi-square (CMIN), CMIN/degree of freedom (DF), *P* value, normed fit index (NFI), incremental fit index (IFI), Tucker–Lewis index (TLI), comparative fit index (CFI), root mean square error of approximation (RMSEA), and standardized root mean residual (SRMR), and values of CMIN/DF < 3, *P* > 0.05, NFI > 0.9, IFI > 0.9, TLI > 0.9、CFI > 0.9, RMSEA < 0.08, and SRMR < 0.08 indicated a good fit^17,18^. *P* < 0.05 was considered statistically significant. The flow chart of the whole study is shown in Fig. 1.

### Ethical standards

This study was approved by the Medical Ethics Committee of the Bengbu Medical College ([2018] No. 015). All experiments were performed in accordance with relevant guidelines and regulations.

### Consent to participate

All individual participants signed the informed consent form, and the neonates' informed consent was signed by their mothers in this study.

### Consent to publish

Patients signed informed consent regarding publishing their data and photographs.

## Results

### Comparisons of prenatal androgens and cord blood androgens between male and female neonates

There were 342 pregnant women in this study, of which the average age at delivery was 28.3 years old. There were 194 male neonates (56.7%) and 148 female neonates (43.3%). The average birth weight was 3.42 kg in male neonates and 3.34 kg in female neonates. The concentrations of cord blood T (CB-T), cord blood FT (CB-FT) and cord blood DHT (CB-DHT) in male neonates were higher than those in female neonates (*P* < 0.05). However, there were no significant differences in the concentrations of prenatal T (P-T), prenatal FT (P-FT), prenatal DHT (P-DHT), prenatal DHEA (P-DHEA), prenatal SHBG (P-SHBG), cord blood DHEA (CB-DHEA), or cord blood SHBG (CB-SHBG) between male and female neonates (*P* > 0.05). The results are shown in Table 1.

**Table 1:** Comparisons of prenatal androgens and cord blood androgens between male and female neonates.

Variables	Male neonates	Female neonates	*t*	*p*	95% CI	
	(n = 194)	(n = 148)				
P-T (ng/dl)	132.6 (73.1)	130.6 (91.0)	0.23	0.819	− 15.826	19.984
P-FT (pg/ml)	2.5 (2.6)	2.3 (1.0)	1.12	0.264	− 0.196	0.715
P-DHT (pg/ml)	51.2 (35.9)	50.6 (33.7)	0.18	0.861	− 7.013	8.387
P-DHEA (ng/ml)	1.2 (4.4)	0.9 (0.9)	0.75	0.456	− 0.455	1.011
P-SHBG (nmol/l)	460.5 (111.5)	481.5 (125.8)	− 1.59	0.113	− 46.915	5.020
CB-T (ng/dl)	136.7 (66.0)	121.6 (52.8)	2.23	0.027	1.755	28.474
CB-FT (pg/ml)	5.3 (2.0)	4.6 (1.6)	3.31	0.001	0.282	1.107
CB-DHT (pg/ml)	35.2 (11.9)	32.1 (10.2)	2.42	0.016	0.567	5.498
CB-DHEA (ng/ml)	1.5 (2.8)	1.5 (2.2)	0.28	0.78	− 0.481	0.641
CB-SHBG (nmol/l)	34.0 (33.8)	37.8 (56.4)	− 0.75	0.455	− 13.678	6.148

*P-T* prenatal testosterone, *P-FT* prenatal free testosterone, *P-DHT* prenatal dihydrotestosterone, *P-DHEA* prenatal dehydroepiandrosterone, *P-SHBG* prenatal sex hormone binding globulin, *CB-T* cord blood testosterone, *CB-FT* cord blood free testosterone, *CB-DHT* cord blood dihydrotestosterone, *CB-DHEA* cord blood dehydroepiandrosterone, *CB-SHBG* cord blood sex hormone binding globulin.

### Comparisons of prenatal androgens and cord blood androgens

In male neonates, the concentration of P-FT was lower than that of CB-FT (*P* < 0.001); however, the concentrations of P-DHT and P-SHBG were higher than those of CB-DHT and CB-SHBG, respectively (*P* < 0.001). In female neonates, the concentrations of P-FT and P-DHEA were lower than those of CB-FT and CB-DHEA, respectively (*P* < 0.001); however, the concentrations of P-DHT and P-SHBG were higher than those of CB-DHT and CB-SHBG, respectively (*P* < 0.001). The results are shown in Table 2.

**Table 2:** Comparisons of differences between prenatal androgens and cord blood androgens.

Variables	Male neonates			Female neonates		
	Mean (SD) of difference	*t*	*P*	Mean (SD) of difference	*t*	*P*
P-T vs CB-T (ng/dl)	− 4.1 (91.1)	− 0.61	0.543	8.9 (91.2)	1.16	0.246
P-FT vs CB-FT (pg/ml)	− 2.8 (1.5)	− 24.62	< 0.001	− 2.3 (1.5)	− 18.38	< 0.001
P-DHT vs CB-DHT (pg/ml)	16.1 (35.3)	6.18	< 0.001	18.4 (33.1)	6.60	< 0.001
P-DHEA vs CB-DHEA (ng/ml)	− 0.4 (5.0)	− 0.98	0.328	− 0.6 (1.6)	− 4.19	< 0.001
P-SHBG vs CB-SHBG (nmol/l)	426.6 (111.3)	51.96	< 0.001	443.7 (137.4)	38.34	< 0.001

*P-T* prenatal testosterone, *P-FT* prenatal free testosterone, *P-DHT* prenatal dihydrotestosterone, *P-DHEA* prenatal dehydroepiandrosterone, *P-SHBG* prenatal sex hormone binding globulin, *CB-T* cord blood testosterone, *CB-FT* cord blood free testosterone, *CB-DHT* cord blood dihydrotestosterone, *CB-DHEA* cord blood dehydroepiandrosterone, *CB-SHBG* cord blood sex hormone binding globulin.

### Association paths between prenatal androgens and cord blood androgens

#### Correlation analysis of prenatal androgens and cord blood androgens

In male neonates, P-T was positively correlated with P-FT, P-DHT, CB-T and CB-FT (*P* < 0.05); P-FT was positively correlated with P-DHT, CB-FT, CB-DHT and CB-SHBG (*P* < 0.05); P-DHT was positively correlated with CB-FT, CB-DHT and CB-DHEA (*P* < 0.05); P-DHEA was positively correlated with CB-DHEA (*P* < 0.05); P-SHBG was negatively correlated with CB-FT (*P* < 0.05); CB-FT was positively correlated with CB-T and CB-DHT (*P* < 0.05); and CB-DHT was positively correlated with CB-DHEA (*P* < 0.05).

**Figure 2:**
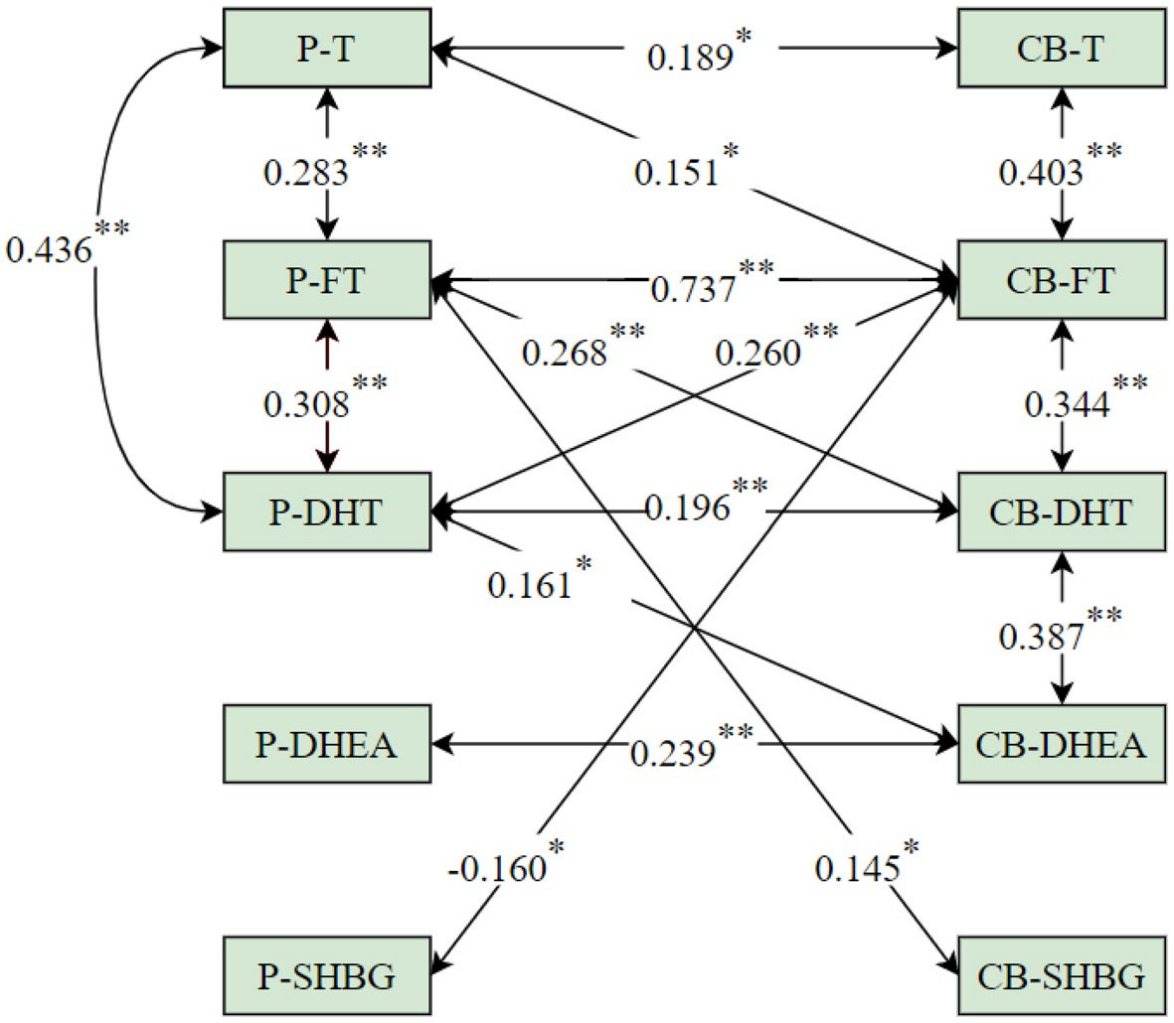
Relationships between prenatal androgens and cord blood androgens in male neonates. Notes: *P-T* prenatal testosterone, *P-FT* prenatal free testosterone, *P-DHT* prenatal dihydrotestosterone, *P-DHEA* prenatal dehydroepiandrosterone, *P-SHBG* prenatal sex hormone binding globulin, *CB-T* cord blood testosterone, *CB-FT* cord blood free testosterone, *CB-DHT* cord blood dihydrotestosterone, *CB-DHEA* cord blood dehydroepiandrosterone, *CB-SHBG* cord blood sex hormone binding globulin; *P < 0.05; **P < 0.01.

**Figure 3:**
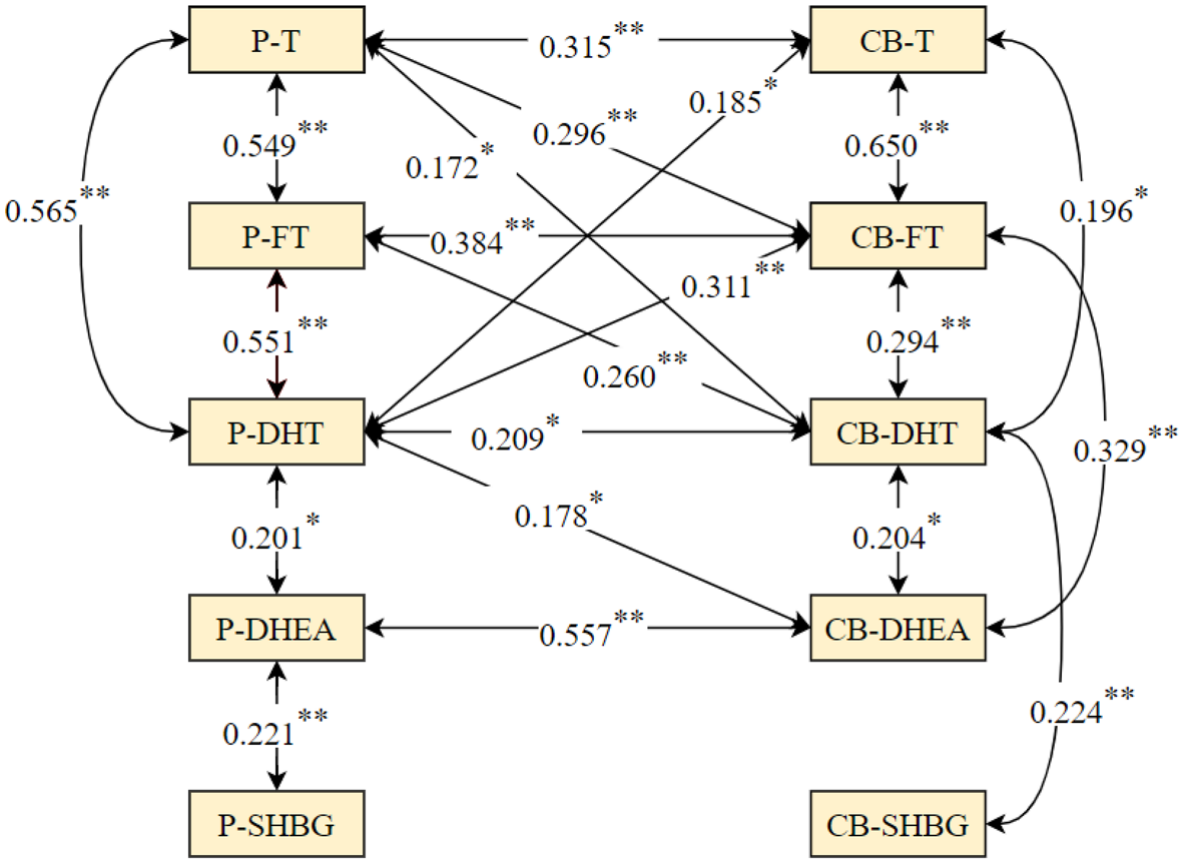
Relationships between prenatal androgens and cord blood androgens in female neonates. Notes: *P-T* prenatal testosterone, *P-FT* prenatal free testosterone, *P-DHT* prenatal dihydrotestosterone, *P-DHEA* prenatal dehydroepiandrosterone, *P-SHBG* prenatal sex hormone binding globulin, *CB-T* cord blood testosterone, *CB-FT* cord blood free testosterone, *CB-DHT* cord blood dihydrotestosterone, *CB-DHEA* cord blood dehydroepiandrosterone, *CB-SHBG* cord blood sex hormone binding globulin; *P < 0.05; **P < 0.01.

Compared to male neonates, in female neonates, positive associations between P-T and CB-DHT, P-DHT and P-DHEA; between P-DHT and CB-T, P-DHEA and P-SHBG; between CB-DHT and CB-T, CB-DHT and CB-SHBG; and between CB-FT and CB-DHEA were found, but no association was found between P-SHBG and CB-FT, P-FT and CB-SHBG. The results are shown in Figs. 2 and 3.

#### Preliminary association path models between prenatal androgens and cord blood androgens

Existing research has shown that T is the source of FT and DHT; FT can also be converted to DHT; DHEA is a precursor of androgens; and T, FT, DHT and DHEA in pregnant women and their newborns may interact with each other through the placental barrier. Combined with the results of the correlation analysis, the following conclusions were drawn. In male neonates, P-FT may be affected by P-T and CB-FT; P-DHT may be affected by P-FT, P-T, CB-FT, CB-DHT and CB-DHEA; P-DHEA may be affected by CB-DHEA; CB-T may be affected by P-T; CB-FT may be affected by CB-T, P-T and P-FT; and CB-DHT may be affected by CB-FT, CB-DHEA, P-FT and P-DHT. In female newborns, P-FT may be affected by P-T and CB-FT; P-DHT may be affected by P-FT, P-T, P-DHEA, CB-T, CB-FT, CB-DHT and CB-DHEA; P-DHEA may be affected by CB-DHEA; CB-T may be affected by P-T; CB-FT may be affected by CB-T, CB-DHEA, P-T and P-FT; and CB-DHT may be affected by CB-FT, CB-DHEA, CB-SHBG, P-T, P-FT and P-DHT.

**Figure 4:**
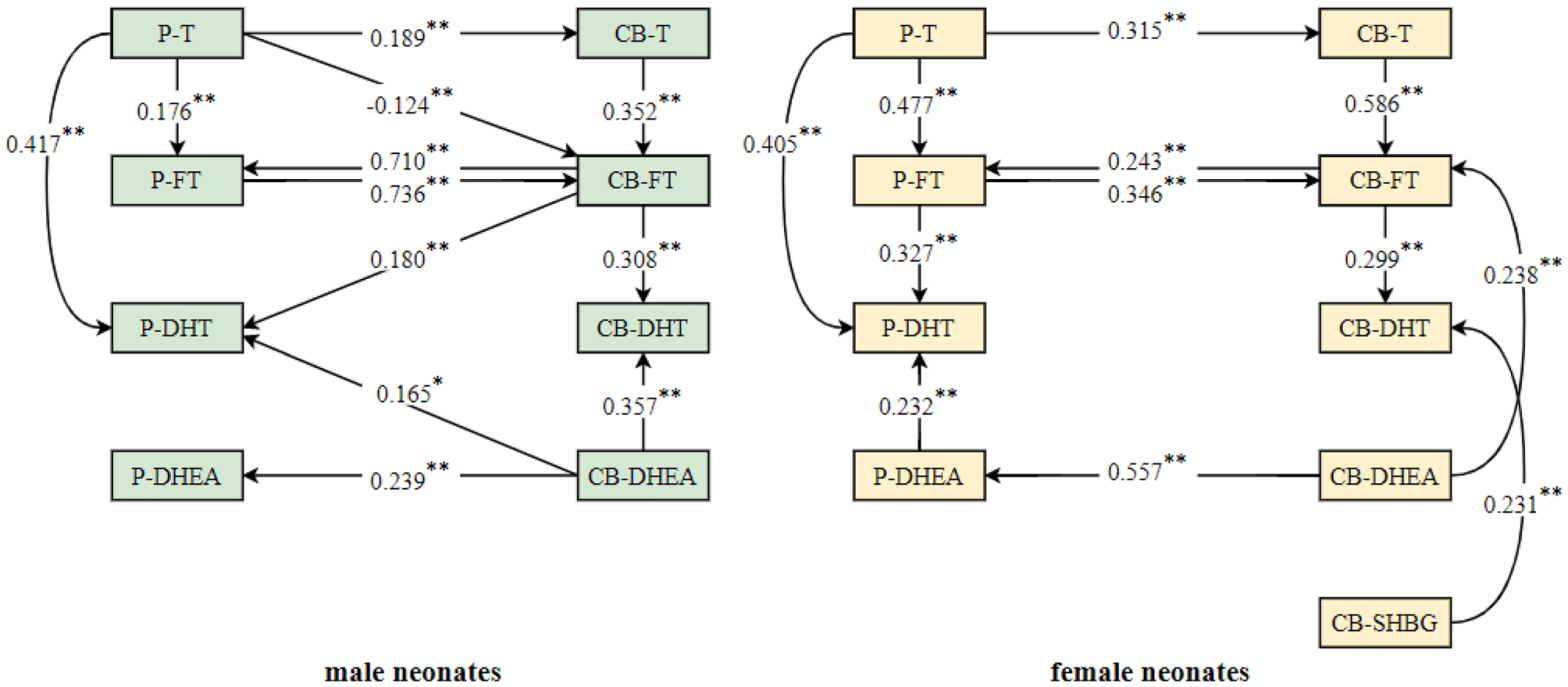
Preliminary association path models between prenatal androgens and cord blood androgens. Notes: *P-T* prenatal testosterone, *P-FT* prenatal free testosterone, *P-DHT* prenatal dihydrotestosterone, *P-DHEA* prenatal dehydroepiandrosterone, *CB-T* cord blood testosterone, *CB-FT* cord blood free testosterone, *CB-DHT* cord blood dihydrotestosterone, *CB-DHEA* cord blood dehydroepiandrosterone, *CB-SHBG* cord blood sex hormone binding globulin; *P < 0.05; **P < 0.01.

Based on the above relations, multiple linear regression analysis was performed and showed that in male neonates, P-FT was related to P-T and CB-FT; P-DHT was related to P-T, CB-FT and CB-DHEA; P-DHEA was related to CB-DHEA; CB-T was related to P-T; CB-FT was related to CB-T, P-T and P-FT; and CB-DHT was related to CB-FT and CB-DHEA. In female neonates, P-FT was related to P-T and CB-FT; P-DHT was related to P-FT, P-T and P-DHEA; P-DHEA was related to CB-DHEA; CB-T was related to P-T; CB-FT was related to CB-T, CB-DHEA and P-FT; and CB-DHT was related to CB-FT and CB-SHBG. The preliminary relationship path between prenatal androgens and cord blood androgens is shown in Fig. 4.

#### Fitting of association path models between prenatal androgens and cord blood androgens

**Figure 5:**
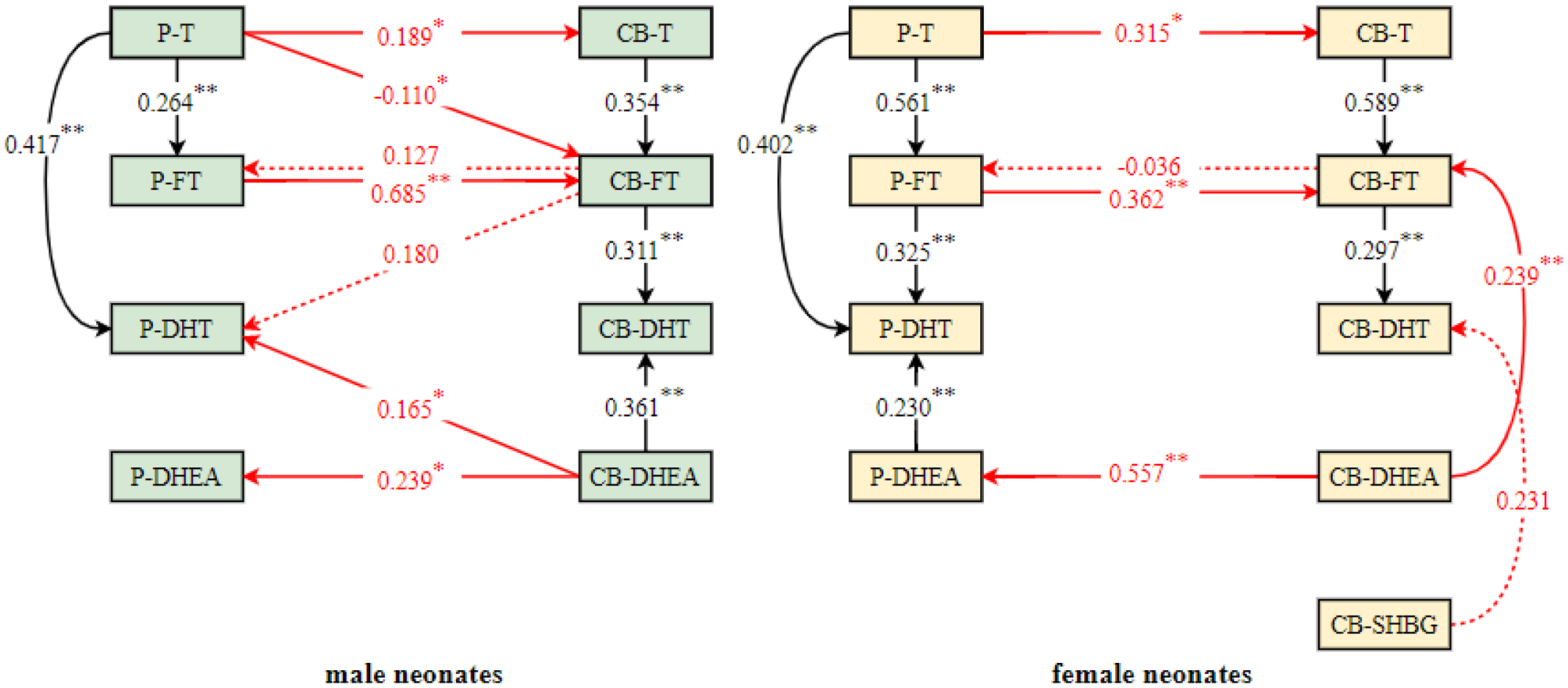
Association path models between prenatal androgens and cord blood androgens. Notes: *P-T* prenatal testosterone, *P-FT* prenatal free testosterone, *P-DHT* prenatal dihydrotestosterone, *P-DHEA* prenatal dehydroepiandrosterone, *CB-T* cord blood testosterone, *CB-FT* cord blood free testosterone, *CB-DHT* cord blood dihydrotestosterone, *CB-DHEA* cord blood dehydroepiandrosterone, *CB-SHBG* cord blood sex hormone binding globulin; Black arrow, already known data; Red arrow, new findings data; *P < 0.05; **P < 0.01.

The preliminary models in male neonates were fitted by path analysis, and the fitting indices were CMIN = 22.323, CMIN/DF = 1.395, P = 0.133, NFI = 0.938, IFI = 0.982, TLI = 0.967, CFI = 0.981, RMSEA = 0.046, and SRMR = 0.0368, which showed that the model had a good fit. The following association paths were found: ① P-T → CB-T → CB-FT → CB-DHT; ; ② P-T → CB-FT → CB-DHT; ③ P-T → P-FT → CB-FT → CB-DHT; ④ P-T → P-DHT; ⑤ CB-DHEA → CB-DHT; ⑥ CB-DHEA → P-DHT; and ⑦ CB-DHEA → P-DHEA. The pathway coefficients for the paths from CB-FT to P-FT and from CB-FT to P-DHT were not significant. The results are shown in Fig. 5 and Table 3.

**Table 3:** Coefficients of association path models between prenatal androgens and cord blood androgens in male neonates.

Paths	β	SE	*P*	95% CI	
**Direct paths**					
P-T → CB-T	0.189	0.104	0.040	0.012	0.399
P-T → CB-FT	− 0.110	0.050	0.024	− 0.225	− 0.022
P-T → P-FT	0.264	0.110	0.003	0.140	0.516
P-T → P-DHT	0.417	0.088	0.001	0.249	0.588
CB-T → CB-FT	0.354	0.095	0.004	0.215	0.547
CB-FT → P-FT	0.127	0.108	0.202	− 0.093	0.344
CB-FT → P-DHT	0.180	0.109	0.087	− 0.017	0.388
CB-FT → CB-DHT	0.311	0.143	0.004	0.061	0.592
P-FT → CB-FT	0.685	0.178	< 0.001	0.263	0.835
CB-DHEA → P-DHEA	0.239	0.115	0.013	0.048	0.472
CB-DHEA → P-DHT	0.165	0.072	0.019	0.024	0.305
CB-DHEA → CB-DHT	0.361	0.139	0.001	0.093	0.622
**Indirect paths**					
P-T → CB-T → CB-FT → CB-DHT	0.021	0.013	0.010	0.004	0.060
P-T → CB-FT → CB-DHT	− 0.034	0.020	0.024	− 0.084	− 0.002
P-T → P-FT → CB-FT → CB-DHT	0.056	0.026	0.002	0.010	0.108
P-T → CB-T → CB-FT → P-DHT	0.012	0.010	0.059	0.000	0.043
P-T → CB-FT → P-DHT	− 0.020	0.014	0.077	− 0.055	0.001
P-T → P-FT → CB-FT → P-DHT	0.033	0.020	0.058	− 0.001	0.074
P-T → CB-T → CB-FT → P-FT	0.009	0.011	0.157	− 0.006	0.036
P-T → CB-FT → P-FT	− 0.014	0.014	0.122	− 0.045	0.004

*P-T* prenatal testosterone, *P-FT* prenatal free testosterone, *P-DHT* prenatal dihydrotestosterone, *P-DHEA* prenatal dehydroepiandrosterone, *CB-T* cord blood testosterone, *CB-FT* cord blood free testosterone, *CB-DHT* cord blood dihydrotestosterone, *CB-DHEA* cord blood dehydroepiandrosterone.

**Figure 6:**
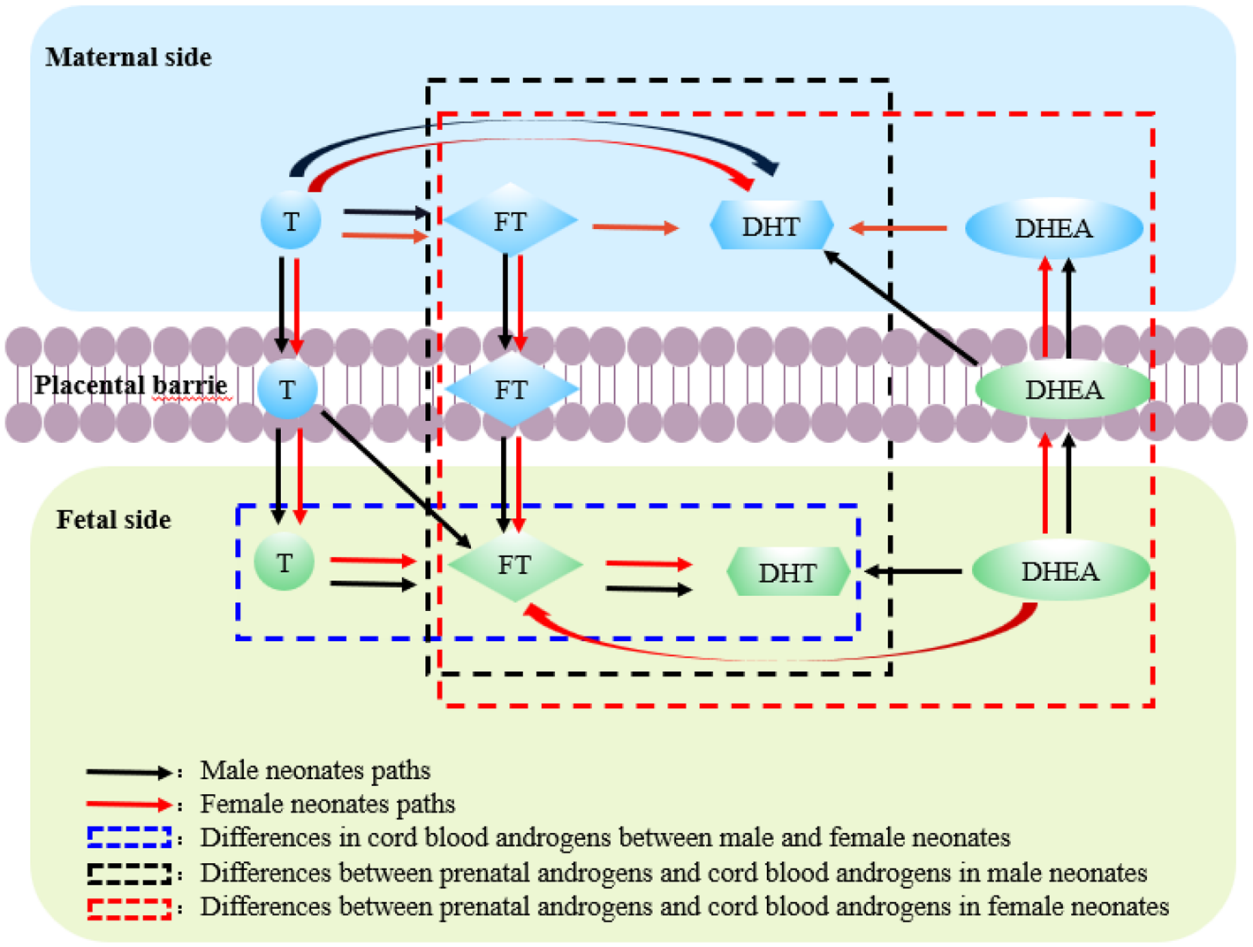
The graphic abstract of association between prenatal androgens and cord blood androgens. Notes: *T* testosterone, *FT* free testosterone, *DHT* dihydrotestosterone, *DHEA* dehydroepiandrosterone; Black arrow, male neonates paths; Red arrow, female neonates paths; Blue dotted frame, differences in cord blood androgens between male and female neonates; Black dotted frame, differences between prenatal androgens and cord blood androgens in male neonates; Red dotted frame, differences between prenatal androgens and cord blood androgens in female neonates.

The preliminary model in female neonates was fitted by path analysis, and the fitting indices were CMIN = 29.615, CMIN/DF = 1.234, P = 0.198, NFI = 0.922, IFI = 0.984, TLI = 0.975, CFI = 0.984, RMSEA = 0.041, and SRMR = 0.0579, which showed that the model had a good fit. The following association paths were found: ① P-T → CB-T → CB-FT → CB-DHT; ② P-T → P-FT → CB-FT → CB-DHT; ③ P-T → P-FT → P-DHT; ④ P-T → P-DHT; ⑤ P-DHEA → P-DHT; ⑥ CB-DHEA → P-DHEA; and ⑦ CB-DHEA → CB-FT. The pathway coefficients for the paths from CB-FT to P-FT and from CB-SHBG to CB-DHT were not significant. The results are shown in Fig. 5 and Table 4. The graphic abstract of association between prenatal androgens and cord blood androgens is shown in Fig. 6.

**Table 4:** Coefficients of association path models between prenatal androgens and cord blood androgens in female neonates.

Paths	β	SE	*P*	95% CI	
**Direct paths**					
P-T → CB-T	0.315	0.138	0.028	0.033	0.560
P-T → P-FT	0.561	0.087	0.001	0.401	0.752
P-T → P-DHT	0.402	0.105	0.001	0.191	0.609
CB-DHEA → CB-FT	0.239	0.074	0.001	0.106	0.411
CB-DHEA → P-DHEA	0.557	0.111	0.003	0.327	0.767
CB-T → CB-FT	0.589	0.053	0.002	0.462	0.678
CB-FT → P-FT	− 0.036	0.103	0.833	− 0.214	0.190
CB-FT → CB-DHT	0.297	0.114	0.006	0.080	0.510
P-FT → CB-FT	0.362	0.074	0.001	0.215	0.497
P-FT → P-DHT	0.325	0.119	0.007	0.105	0.573
CB-SHBG → CB-DHT	0.231	0.114	0.060	− 0.019	0.425
P-DHEA → P-DHT	0.230	0.076	0.001	0.096	0.395
**Indirect paths**					
P-T → CB-T → CB-FT → CB-DHT	0.055	0.039	0.021	0.004	0.158
P-T → P-FT → CB-FT → CB-DHT	0.060	0.034	0.003	0.014	0.156
P-T → CB-T → CB-FT → P-FT	− 0.007	0.020	0.570	− 0.050	0.035
P-T → P-FT → P-DHT	0.182	0.073	0.004	0.069	0.366
CB-DHEA → P-DHEA → P-DHT	0.128	0.057	0.002	0.042	0.274

*P-T* prenatal testosterone, *P-FT* prenatal free testosterone, *P-DHT* prenatal dihydrotestosterone, *P-DHEA* prenatal dehydroepiandrosterone, *CB-T* cord blood testosterone, *CB-FT* cord blood free testosterone, *CB-DHT* cord blood dihydrotestosterone, *CB-DHEA* cord blood dehydroepiandrosterone, *CB-SHBG* cord blood sex hormone binding globulin.

## Discussion

The association paths between prenatal androgens and cord blood androgens are complex. Revealing the specific correlation paths is of great significance to promote newborn health. This study showed that CB-T, CB-FT and CB-DHT concentrations in male neonates were higher than those in female neonates. In male and female neonates, the concentration of P-FT was lower than that of CB-FT and the concentrations of P-DHT and P-SHBG were higher than those of CB-DHT and CB-SHBG, respectively. In female newborns, the concentration of P-DHEA was lower than that of CB-DHEA. The association paths between prenatal androgens and cord blood androgens were determined, and the concentrations of P-T and P-FT were positively associated with those of CB-T and CB-FT, while the concentration of CB-DHEA was positively associated with that of P-DHEA.

The results of this study showed that the concentrations of CB-T, CB-FT and CB-DHT in male neonates were higher than those in female neonates. This might be related to the fact that female fetuses are considered to be endocrine inert, while male fetuses have an established feedback system at the hypothalamic–pituitary–gonadal (HPG) axis^19,20^. The results of this study showed that the concentration of P-FT was lower than that of CB-FT in male and female neonates. Leslie et al.^21^ reported that the activity of placental aromatase decreases with the process of delivery, and the conversion of androgen to estrogen in the placenta decreases accordingly, which leads to an increase in CB-FT. The results of this study showed that the concentrations of P-DHT and P-SHBG were higher than those of CB-DHT and CB-SHBG. Placental expression of 5α-reductase increases with advancing gestation^22^, and the conversion of T to DHT increases, which leads to an increase in P-DHT. However, the P-SHBG concentration was higher than that of CB-SHBG, possibly because estradiol stimulates the maternal liver to produce a large amount of SHBG^16^. The results of this study showed that the concentration of P-DHEA was lower than that of CB-DHEA in female neonates. Some studies have shown that placental corticotropin-releasing hormone increases exponentially in late pregnancy, thus promoting the release of CB-DHEA^23–25^ the much higher concentration of CB-DHEA compared to that of P-DHEA observed in female neonates may also be related to the limited activity of 16-hydroxylase in the maternal liver^16^, while the strong activities of both 17-hydroxylase and 17–20 lyase expressed in the inner zone of the fetal adrenal gland abundantly produce DHEA(S)^26^.

In male neonates, path analysis showed that the concentrations of T, FT, DHT and DHEA in prenatal venous blood were associated with those of T, FT, DHT and DHEA in cord blood, and the following pathways were identified: ① P-T → CB-T → CB-FT → CB-DHT; ② P-T → CB-FT → CB-DHT; ③ P-T → P-FT → CB-FT → CB-DHT; ④ P-T → P-DHT; ⑤ CB-DHEA → CB-DHT; ⑥ CB-DHEA → P-DHT; and ⑦ CB-DHEA → P-DHEA. In this study, P-T was shown to be converted to P-FT and P-DHT, and P-T and P-FT concentrations were shown to affect CB-T and CB-FT concentrations, indicating that P-T and P-FT are fat-soluble steroids that can cross the placental barrier^27,28^. CB-FT was generated from P-T, P-FT, and CB-T and was further converted to CB-DHT in male neonates, And most studies^27–29^ have found correlations between them. P-FT has been reported to be correlated with CB-FT in males but not in females^30^. A study by Ahrenfeldt et al.^11^ showed that maternal–fetal hormone transfer may be unidirectional, with passage of hormones only from the mother to the fetus. Clearly, the findings of this study support this idea. The results of this study showed that CB-DHEA was not only converted into CB-DHT but also crossed the placenta into prenatal venous blood, and the concentration of CB-DHEA was correlated with those of P-DHEA and P-DHT, it may be that the level of DHEA produced on the fetal side is higher than that on the maternal side^26^. Nieschlag^31^ found that DHEA originating from fetal DHEAS was not entirely converted to estrogens but was also released into maternal circulation. Some studies^29^ have shown that there is a significant correlation between the concentrations of P-DHEA and CB-DHEA. However, Goolsby^32^ showed that there was no correlation between the concentrations of P-DHEA and CB-DHEA, which was inconsistent with the results of this study.

In female neonates, path analysis showed that P-T, P-FT, P-DHT and P-DHEA were associated with CB-T, CB-FT, CB-DHT and CB-DHEA. The following pathways have the same structure in female neonates as in male neonates: ① P-T → CB-T → CB-FT → CB-DHT; ② P-T → P-FT → CB-FT → CB-DHT; ③ P-T → P-DHT; and ④ CB-DHEA → P-DHEA. The following additional paths were observed in female neonates: P-FT → P-DHT, P-DHEA → P-DHT, and CB-DHEA → CB-FT; and the following paths in male neonates were not observed in female neonates: P-T → CB-FT, CB-DHEA → P-DHT, and CB-DHEA → CB-DHT. There were sex differences in the P-T → CB-FT, P-FT → P-DHT and CB-DHEA → P-DHT paths, which may be due to the differential expression of enzymes in the placentas of male and female neonates. Numerous proteins involved in androgen biosynthesis and signaling have been identified in the human placenta, and the expression and activity of these proteins may be altered by fetal-placental sex^33,34^. In addition, the level of P-FT may be insufficient to allow for its conversion to P-DHT due to the greater amount of conversion to CB-FT through the placenta in male neonates, while in female neonates, P-FT may be converted to P-DHT to prevent excessive transfer of FT from prenatal blood to cord blood. There were sex differences in the P-DHEA → P-DHT path, which may be because the rate of conversion of P-DHEA to estrogen is greater in the placenta and the amount of P-DHEA released from the maternal adrenal gland is less than that released by the fetal adrenal gland, resulting in insufficient transformation of P-DHEA to P-DHT in male neonates. Path analysis showed that CB-DHT was converted from CB-FT and CB-DHEA in male neonates, while the path from CB-DHEA to CB-DHT was not found in female neonates, indicating that the concentration of CB-DHT in female neonates was lower than that in male neonates^35^. At the same time, some studies have found that DHT synthesis occurs not only in the testis and adrenal gland but also in the human ovary^36,37^. There was a correlation between the concentrations of CB-DHEA and CB-FT in female neonates but not in male neonates, which may be because DHEA is regulated by the HPA axis, while FT is mainly regulated by the HPG axis. There is evidence^38,39^ of an interaction between the HPA axis and HPG axis, and there are sex differences in this interaction. Human studies^40,41^ have shown that prenatal exposure to high levels of fetal T results in the masculinization of the brain. This phenomenon was first observed in girls with congenital adrenal hyperplasia, which indicated that the HPA axis may have a regulatory effect on the HPG axis, and this effect is stronger in girls than in boys.

No association between SHBG and androgens was found in these structural paths, which was in line with the results from a large birth cohort^42^. Under normal conditions, SHBG can bind to and transport T and DHT. The concentration of SHBG increased from 5 to 10-fold during mid to late pregnancy, which changed the affinity of SHBG for T and DHT^43^. Heinrich-Balard et al.^44^ revealed an inverse relationship between SHBG affinity for T. In addition, the increase in estrogen in late pregnancy was much higher than the increases in T and DHT, and estrogen may compete with T and DHT for the available binding sites of SHBG^45^.

This study has some limitations. First, this study was cross-sectional; thus, the causal relationship between prenatal androgens and cord blood androgens could not be determined, and the results need to be verified in cohort studies. Second, the level of sex hormones in cord blood may be affected by adrenal activation and delivery. Finally, other potential confounding factors were not taken into account in this study.

## Conclusions

We found differences between androgen concentrations in prenatal blood and cord blood and differences in the concentrations of prenatal androgens and cord blood androgens between male and female neonates and developed association paths between prenatal androgens and cord blood androgens. The association paths showed that P-T and P-FT can pass through the placental barrier positively affecting the concentrations of CB-T and CB-FT, while CB-DHEA can pass through the placental barrier positively affecting the concentration of P-DHEA. These findings are of great significance for understanding the interactions of maternal androgens and neonatal androgens and providing a theoretical basis for reducing the adverse birth outcome of newborns and promoting the early prevention of childhood diseases. Of course, the specific mechanism needs to be verified in cohort studies or experimental studies.

## Data Availability

The datasets are available from the corresponding author on reasonable request.

## Abbreviations

CB-DHEA: Cord blood dehydroepiandrosterone
CB-DHT: Cord blood dihydrotestosterone
CB-FT: Cord blood free testosterone
CB-SHBG: Cord blood sex hormone binding globulin
CB-T: Cord blood testosterone
CFI: Comparative fit index
CMIN: Chi-square
DF: Degree of freedom
DHT: Dihydrotestosterone
DHEA: Dehydroepiandrosterone
DHEAS: Dehydroepiandrosterone sulfate
FT: Free testosterone
HPA: Hypothalamic–pituitary–adrenal
HPG: Hypothalamic–pituitary–gonadal
IFI: Incremental fit index
NFI: Normed fit index
P-DHEA: Prenatal dehydroepiandrosterone
P-DHT: Prenatal dihydrotestosterone
P-FT: Prenatal free testosterone
P-T: Prenatal testosterone
P-SHBG: Prenatal sex hormone binding globulin
RMSEA: Root Mean Square Error of Approximation
SHBG: Sex hormone binding globulin
SRMR: Standardized Root Mean Residual
T: Testosterone
TLI: Tucker–Lewis index
